# Comparison of Ex-PRESS implantation versus trabeculectomy combined with phacoemulsification in primary open-angle glaucoma: a retrospective in vivo confocal microscopy study

**DOI:** 10.1186/s40662-022-00278-2

**Published:** 2022-02-10

**Authors:** Yuqiao Zhang, Beiting He, Yulin Zhang, Jin Zeng, Yanlei Chen, Yongyi Niu, Honghua Yu, Yongjie Qin, Hongyang Zhang

**Affiliations:** 1 Department of Ophthalmology, Guangdong Eye Institute, Guangdong Academy of Medical Sciences, Guangdong Provincial People’s Hospital, No. 106 Zhongshan Er Road, Guangzhou, 510080 China; 2 Shantou University Medical College, Shantou, China; 3 The Second School of Clinical Medicine, Southern Medical University, Guangzhou, China; 4 Department of Ophthalmology, School of Medicine, South China University of Technology, Guangzhou, China

**Keywords:** POAG, Ex-PRESS, Phacoemulsification, Confocal microscopy

## Abstract

**Background:**

To compare the efficacy of Ex-PRESS implantation versus trabeculectomy combined with phacoemulsification.

**Methods:**

A retrospective 12-month study on patients with coincident primary open-angle glaucoma (POAG) and cataract. The patients underwent combined phacoemulsification and Ex-PRESS implant (Phaco-ExPRESS, n = 35) or phacotrabeculectomy (Phaco-Trab, n = 35). The morphological structures of the filtering bleb were examined by slit-lamp, anterior segment optical coherence tomography (AS-OCT) and in vivo confocal microscopy (IVCM). Complete success was defined as postoperative intraocular pressure (IOP) < 18 mmHg without the use of anti-glaucoma medication. Qualified success was defined as postoperative IOP < 18 mmHg with or without anti-glaucoma medications. The data were collected preoperatively and postoperatively at 2 weeks, 1 month, 3 months, 6 months, and 12 months.

**Results:**

No significant difference in the variables such as age, IOP and perimetry was found between the groups of Phaco-ExPRESS and Phaco-Trab. At the one-year postoperative visit for filtering blebs, Phaco-ExPRESS increased the mean area of epithelial microcysts significantly from 0.10 ± 0.05 to 0.20 ± 0.09 μm^2^ per μm^2^, while Phaco-Trab decreased the mean area significantly from 0.08 ± 0.04 to 0.04 ± 0.06 μm^2^ per μm^2^. Notably, the hyperreflective dots detected by IVCM decreased by 84.9% in Phaco-ExPRESS but increased by 36.3% in Phaco-Trab. The hyperreflective dots were further identified as neutrophil- and monocyte-like cells. The number of these cells were negatively correlated with the microcysts area (r =  − 0.7, *P* < 0.01) but positively associated with the grade of connective tissue (r = 0.5, *P* < 0.01). By creating different microstructural changes in the filtering blebs, Phaco-ExPRESS produced a higher complete success rate (84.9% *vs.* 41.2%, *P* < 0.01) and significant decrease in the number of anti-glaucoma medications (*P* < 0.01) when compared with those in Phaco-Trab. However, the qualified success showed no significant difference between the two groups (100.0% vs. 91.2%, *P* = 0.24).

**Conclusions:**

At the one-year follow-up, Phaco-ExPRESS generated better filtering bleb with larger area of microcysts, looser connective tissues, and less inflammation than that of Phaco-Trab, providing adequate IOP control and less IOP-lowering medications. These findings indicate that Phaco-ExPRESS could be more preferred than Phaco-Trab for the treatment of patients with coincident POAG and cataract.

**Supplementary Information:**

The online version contains supplementary material available at 10.1186/s40662-022-00278-2.

## Background

The management of coincident cataract and primary open-angle glaucoma (POAG) is a common clinical challenge [1]. Phacoemulsification alone can result in intraocular pressure (IOP) decrease by around 1 mmHg for patients with POAG [2, 3], and they may need subsequent filtering surgery. Trabeculectomy is an effective surgical procedure to reduce IOP, but it leads to the progression of cataract [4], and cataract extraction may deteriorate the morphology and function of filtering bleb [5, 6]. Thus, it is practical to perform combined phacoemulsification and trabeculectomy (Phaco-Trab) to treat both diseases in one setting [7]. However, Phaco-Trab was not as potent as trabeculectomy alone in lowering IOP [8]. It is possible that combined procedure amplified the anterior chamber inflammation and increased the chance of filtration bleb scarring, leading to poorer long-term filtration [8, 9]. Currently, there is a lack of consensus regarding the best surgical treatment for coincident POAG and cataract. 

Trabeculectomy has become the gold standard of filtering surgery which is widely used in managing patients with glaucoma. The Ex-PRESS glaucoma minishunt (Alcon, Fort Worth, TX, USA) is a stainless-steel glaucoma device [10]. Its implantation can create a conjunctival bleb similar to trabeculectomy and showed equal effectiveness as trabeculectomy in IOP reduction [10–12]. Notably, Ex-PRESS glaucoma minishunt implantation induces less tissue traumatization that potentially reduce postoperative inflammation [13, 14], which suggests that Ex-PRESS implantation might be an alternative for the combined surgery. Efficacy of maintaining long term IOP reduction and decrease in the number of anti-glaucoma medications has been shown in combined Ex-PRESS implant and phacoemulsification [15]. However, only one study has compared the effectiveness of Ex-PRESS implant and trabeculectomy combined with phacoemulsification [16]. Therefore, further study is needed.

Bleb morphology is an important indicator of bleb function which predicts the outcome of surgery. However, the morphology changes of the blebs after Ex-PRESS implant and trabeculectomy combined with phacoemulsification remain to be identified. As the assessment of filtering blebs by slit-lamp is subjective and insufficient, in vivo confocal microscopy (IVCM) has been proposed to evaluate the functioning and failed bleb at the cellular levels [17–19]. We therefore used IVCM to compare cellular morphologies of the filtering bleb between Ex-PRESS implantation combined with phacoemulsification (Phaco-ExPRESS) and Phaco-Trab, aiming to determine the effectiveness of the two combined surgical management for the patients with coincident POAG and cataract.

## Methods

This was a retrospective study, which was carried out in accordance with the tenets of the Declaration of Helsinki. The study enrolled consecutive Chinese patients who visited the glaucoma clinic of Guangdong Provincial People’s Hospital between November 2019 and March 2021. Informed consent was obtained from all participants.

Inclusion criteria: (1) Diagnosis of POAG and best-corrected visual acuity (BCVA) > 0.1 logMAR; (2) Uncontrolled IOP (> 20 mmHg, mean of three measurements at 9 a.m., 12 p.m., and 4 p.m.) under maximal tolerated medical therapy (unmodified during the last 3 months); (3) Progression of glaucomatous damage confirmed on three consecutive visual fields (VF; 24–2 test, full-threshold, Humphrey field analyzer II 750; Carl Zeiss Meditec, Inc., Dublin, CA, USA). Exclusion criteria were: (1) Diagnosis of angle-closure, neovascular or uveitic glaucoma; (2) History of intraocular surgery; systemic or topical therapies other than anti-glaucoma in the last 6 months or the ocular surface contact lens wearing. (3) Patients who had other ocular diseases, including keratitis, corneal decompensation, retinal detachment, etc. (4) Preoperative active inflammatory status of the ocular anterior segment.

The following data were collected preoperatively and 2 weeks, 1 month, 3 months, 6 months and 12 months postoperatively from each patient: BCVA, central corneal thickness (SW-1000P, Suowei, China), IOP (measured by Goldmann applanation tonometry), slit-lamp anterior segment photography (BX-900, HAAG-STREIT, USA), gonioscopy (O2M, Ocular, USA) using the SCHEIE system, fundus color stereo photography (Canon, Tokyo, Japan), standard automated perimetry (Humphrey Field Analyzer II 750; 24-2 Swedish interactive threshold algorithm, Carl Zeiss Meditec, Dublin, CA, USA), optical biometry measurement (Lenstar LS900, Haag-Streit, USA), spectral domain optical coherence tomography (Spectralis, Heidelberg Engineering GmbH, Heidelberg, Germany), anterior segment optical coherence tomography (AS-OCT) (RTVue-XR Avanti; Optovue, Fremont, CA, USA, version 2016.2.035) and in vivo confocal microscope (HRT III Rostok Cornea Module; Heidelberg Engineering, Heidelberg, Germany).

Glaucoma severity was staged based on the standard automated perimetry using the Hodapp-Parrish-Anderson criteria [20]: mild glaucomatous damage was defined as mean deviation (MD): ≥ − 6 dB, less than 25% of points are depressed < 5% and less than 10 points are depressed < 1% on a pattern deviation plot, all points in central 5° with sensitivity ≥ 15 dB; moderate glaucomatous damage was defined as − 12 dB < MD < − 6 dB, less than 50% of points are depressed < 5% and less than 20 points are depressed < 1% on a pattern deviation plot, only one hemifield having a point in the central 5° with sensitivity < 15 dB, no point within 5° fixation with sensitivity of 0 dB, while severe glaucomatous damage was defined as MD ≤ − 12 dB, more than 50% of points are depressed < 5% and more than 20 points are depressed < 1% on a pattern deviation plot, both hemifield having points in the central 5° with sensitivity < 15 dB, at least one point within 5° fixation with sensitivity of 0 dB.

For all patients, according to recommendations described by Simon et al. [21], complete success was defined as postoperative IOP < 18 mmHg without anti-glaucoma medications. Qualified success was defined as postoperative IOP < 18 mmHg with or without medications.

### Surgery procedure

Patients were divided into two different groups: Phaco-Trab group and Phaco-ExPRESS group. The two different types of surgeries were chosen by patients according to their preferences. All surgeries were performed under topical anesthesia 0.5% proparacaine hydrochloride (Alcaine, Alcon, USA) by a single experienced surgeon. Both procedures included the creation of fornix-based incisions. In both procedures, a 5 × 4 mm, half-thickness scleral flap was created, and small pieces of surgical sponge soaked in 0.4 mg/mL mitomycin C were inserted under the conjunctival flap for 4 min, followed by phacoemulsification of cataract and acrylic intraocular lens (Akreos Advanced Ai, Bausch & Lomb) implantation. In Phaco-ExPRESS, a 25-gauge needle was horizontally inserted into the anterior chamber from the sclera-cornea transition zone parallel with the iris. The Ex-PRESS of model P50 (Alcon, Fort Worth, TX) was then inserted into the anterior chamber. In Phaco-Trab, iridectomy was done after sclerostomy. All the scleral flaps were stitched with two 10–0 nylon sutures and conjunctival flaps were also sutured with 10–0 nylon in both types of surgery.

All eyes that underwent surgery received topical steroids for 4 weeks (dexamethasone 0.2% eye drops four times a day), steroids ointment (dexamethasone 0.2% eye ointment once daily) as well as topical antibiotic for the initial 2 weeks (levofloxacin 5 mg/mL eye drops four times a day). Patients underwent postoperative procedures, such as laser suture lysis, bleb needling and subconjunctival injections of 5-fluorouracil (5-FU) when necessary.

### Assessment of filtering bleb

The micromorphological structures of filtering bleb, including epithelial microcysts, stromal connective tissue and hyperreflective dots were analyzed by in vivo confocal microscope (HRT III Heidelberg Engineering, Germany), which was described previously [22–24]. The IVCM images were obtained from five regions of the filtering bleb in each patient. The mean area of epithelial microcyst and density of hyperreflective dots were calculated by the average value from five regions of the filtering bleb using the ImageJ software. Specimens over the filtering bleb were also obtained by scraping at the filtering bleb conjunctiva and Giemsa stain was used to identify the cell type of hyperreflective dots. Average gray scale of stroma was reviewed for grading of connective tissue. An arbitrary grading scale was adopted to quantify the confocal image reflectivity of the deep stroma. The stromal meshwork reflectivity was calculated by average gray value of the selected image using the ImageJ software. According to Matropasqua et al. [23], this value corresponded to the sum of gray values of all pixels in the entire image divided by the number of pixels. An average gray value less than 90.00 indicated a normal reflectivity (grade 0), from 90.01 to 105.00 mild reflectivity (grade 1), 105.01 to 125.00 moderate (grade 2), and greater than 125.01 high reflectivity (grade 3). Therefore, grades 0 to 3 corresponded to a loosely, mildly dense, dense, and very dense arranged stromal network, respectively (Additional file 1: Fig. S1). Moreover, the filtering blebs were examined by AS-OCT. A single operator performed all AS-OCT and confocal examinations as well as selecting the images, and the other two operators were responsible for evaluating confocal images. Both operators were masked for the grouping and medical history of subjects. They independently evaluated the area of epithelial microcysts, hyperreflective dot density and connective tissue grade to assess the interobserver variability. Each filtering bleb was evaluated at 2 weeks, 1 month, 3 months, 6 months, and 12 months, postoperatively.

To have a better understanding of morphology differences between functioning and nonfunctioning blebs, we measured two typical filtering blebs with AS-OCT, slit-lamp, and in vivo confocal microscope. According to Antoine et al. [17], functioning blebs were defined as IOP < 18 mmHg without anti-glaucoma medications, and nonfunctioning blebs were defined as IOP ≥ 18 mmHg and/or having the need for anti-glaucoma medications.

### Statistical analysis

Analysis was performed using the SPSS Software (SPSS Inc., Chicago, IL, USA). Student’s t-test was used to evaluate baseline differences between groups. A two-way ANOVA was used for multi-comparison of each follow-up time point. The correlations among variables were determined using Spearman’s index. Kaplan-Meier survival analysis was conducted to compare the qualified and complete success between the two groups. Spearman ρ test was utilized to evaluate the correlation between the number of anti-glaucoma medications and the density of hyperreflective dots at 12-month follow-up. A *P* value < 0.05 was considered statistically significant. The value obtained by investigator 1 (YQZ) were used for the statistical analysis, and the values obtained by investigator 2 (BTH) were used to assess the interobserver agreement. Intraclass correlation coefficients (ICC) were also used to evaluate interobserver agreement.

## Results

In total, 70 eyes of 70 patients with POAG were included in Phaco-ExPRESS (n = 35) and Phaco-Trab (n = 35). The average follow-up period was 16.3 ± 3.2 months in Phaco-ExPRESS and 15.2 ± 2.5 months in Phaco-Trab. Two patients in Phaco-ExPRESS and one patient in Phaco-Trab were excluded due to follow-up time less than 12 months. The basic characteristics of patients are summarized in Table 1. No statistically significant difference in these variables was found between the two groups.

**Table 1:** Patients’ characteristics at baseline

	Phaco-ExPRESS (n = 33)	Phaco-Trab (n = 34)	*P* value
Gender (M/F)	17 / 16	18 / 16	0.91
Age (years)	74.76 ± 4.75	72.50 ± 6.86	0.12
MD (dB)	− 14.49 ± 6.21	− 16.44 ± 7.89	0.27
PSD (dB)	7.58 ± 3.22	7.16 ± 3.09	0.58
Mild/moderate/severe (%)	12.1 / 27.3 / 60.6	5.9 / 38.2 / 55.9	0.50
BCVA (logMAR)	0.29 ± 0.18	0.29 ± 0.16	0.10
CCT (mm)	542.24 ± 16.85	545.44 ± 21.53	0.83
C/D ratio	0.84 ± 0.12	0.81 ± 0.11	0.12
Average thickness of GCC (μm)	75.34 ± 8.56	74.86 ± 14.51	0.12
Average thickness of pRNFL (μm)	72.38 ± 10.93	70.81 ± 12.91	0.71
Axial length (mm)	23.84 ± 1.49	23.92 ± 1.13	0.69
Corneal endothelial cell count (cells/mm^2^)	2037.58 ± 262.30	2017.65 ± 260.36	0.76
Preoperative hyperreflective dot count (cells/mm^2^)	0.55 ± 1.33	0.71 ± 1.55	0.40

*M/F* = male/female; *MD* = mean deviation; *PSD* = pattern standard deviation; *Mild/moderate/severe* = mild stage/moderate stage/severe stage of glaucoma; *BCVA* = best-corrected visual acuity; *CCT* = central corneal thickness; *C/D ratio* = cup to disc ratio; *GCC* = ganglion cell complex; *pRNFL* = peripapillary retinal nerve fiber layer. *P* value < 0.05 is considered statistically significant

### Evaluation of the functioning and nonfunctioning filtering blebs

**Fig. 1:**
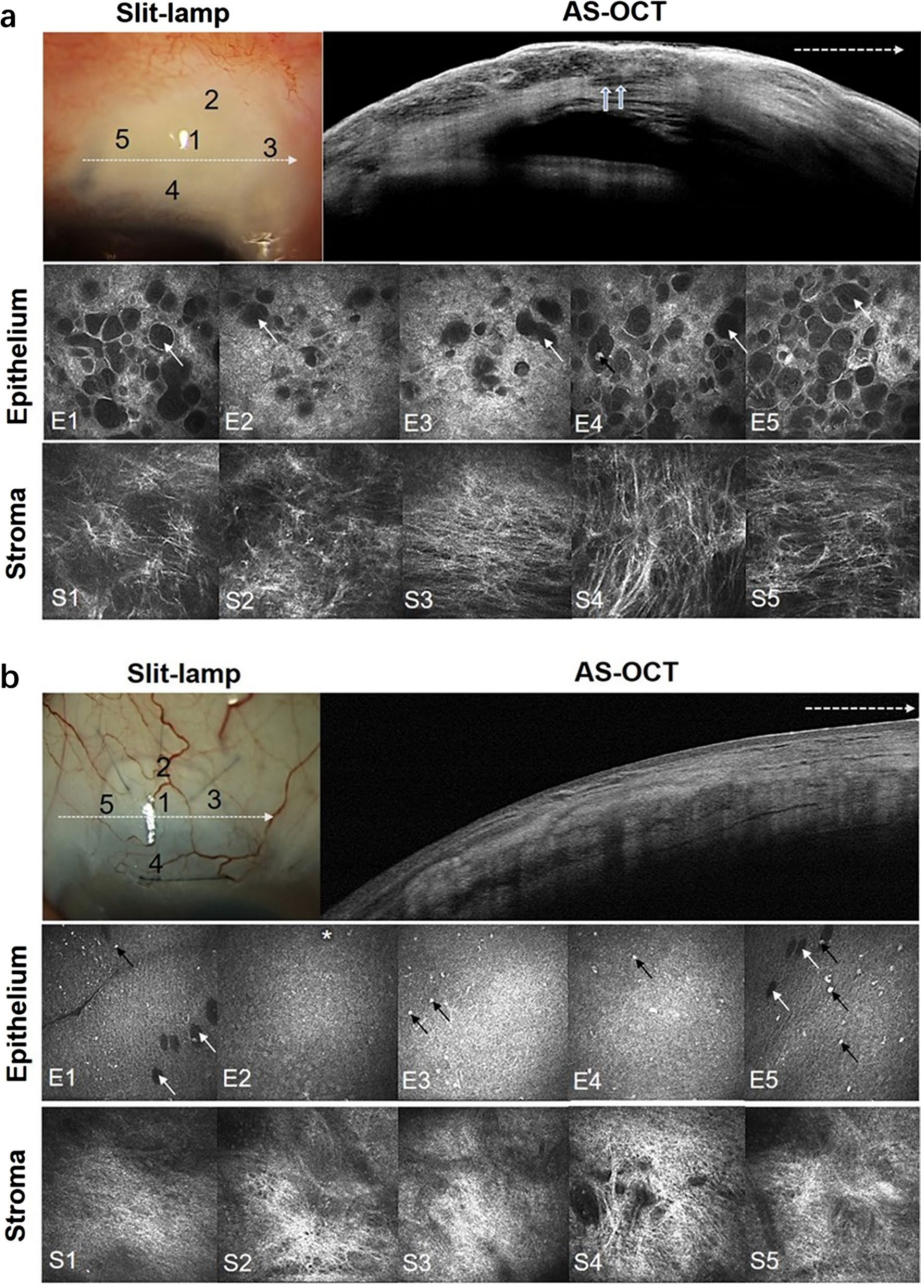
Filtering blebs after trabeculectomy. Functioning and nonfunctioning filtering blebs were visualized by in vivo confocal microscopy (IVCM) at 12 months after trabeculectomy. A filtering bleb can be seen under the slit-lamp. All five sites numbered 1 to 5 in the slit-lamp photograph were taken for IVCM. The anterior segment optical coherence tomography (AS-OCT) was taken across the line showed by white dotted arrow. For each scanning site of IVCM, epithelium and stroma were scanned for evaluation. **a** Elevated filtering bleb with striping phenomenon (multiple parallel hypo-reflective layers inside the presumed Tenon’s layer) (blue arrow) can be seen in AS-OCT. Larger microcysts area (white arrow) and fewer hyperreflective dots (black arrow) were found in epithelium of functioning bleb (E1–E5). Loose connective tissue (S1–S5) was revealed in the functioning bleb. **b** In AS-OCT, the flat filtering bleb and hyperreflective bleb wall can be seen in the nonfunctioning bleb. In IVCM, E1-E5 showed few small microcysts area (white arrow) with scattered hyperreflective dots (black arrow). S1–S5 showed dense connective tissue with hyperreflective fibrous network and corrugated blood vessels

In the functioning filtering bleb (Fig. 1a), a representative cystic space was visible by slit-lamp. IVCM further demonstrated that the cystic space was constituted with numerous microcysts in the epithelium and multiple parallel hyporeflective layers in the stroma. The average connective tissue grade was 0.2. However, in a nonfunctioning bleb (Fig. 1b), the appearance was flat and angioplerotic, constituting numerous hyperreflective dots but less microcysts in the epithelium and mass of hyperreflective networks in the stroma. The average connective tissue grade was 2.4.

### Control of IOP

The average preoperative IOP under maximal tolerated medical therapy were 21.61 ± 5.72 mmHg in Phaco-ExPRESS and 21.60 ± 3.49 mmHg in Phaco-Trab. After surgery, Phaco-ExPRESS showed statistically lower IOP than Phaco-Trab at 2 weeks (9.21 ± 2.59 mmHg vs. 10.91 ± 2.49 mmHg, *P* = 0.01) and 1 month (10.57 ± 2.58 mmHg vs. 12.08 ± 3.03 mmHg, *P* = 0.04). However, no statistical significance was found at 3 months, 6 months, and 12 months postoperatively. At 12 months postoperatively, the IOP was significantly reduced when compared with baseline in both groups (*P* < 0.01).

**Fig. 2:**
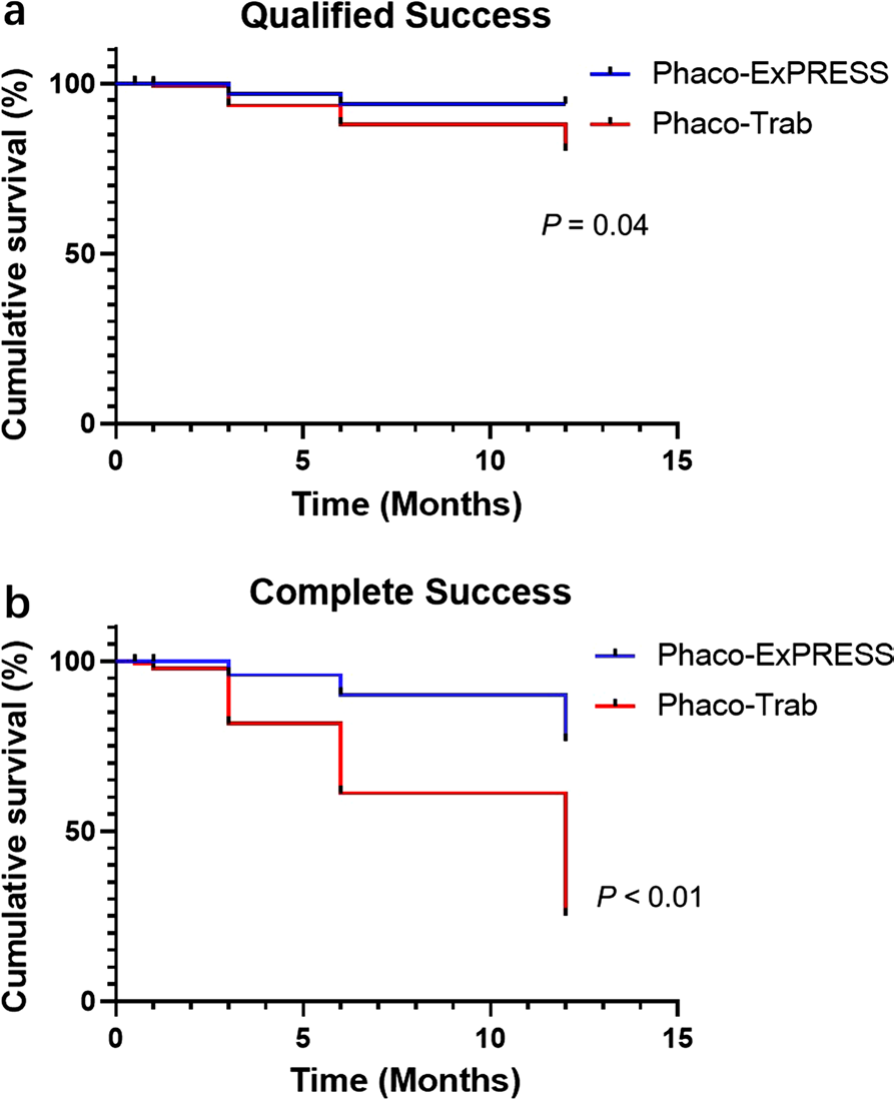
Kaplan-Meier survival analysis. Kaplan-Meier survival analysis of qualified (**a**) and complete (**b**) success. Qualified success was defined as postoperative intraocular pressure (IOP) < 18 mmHg attained with or without anti-glaucoma medication. Complete success was defined as postoperative IOP < 18 mmHg achieved without anti-glaucoma medication. There were statistically significant differences of cumulative probability of success both in qualified and complete success between the Phaco-ExPRESS and Phaco-Trab groups

The mean numbers of preoperative and postoperative anti-glaucoma medications are shown in Table 2. Before surgery, no statistically significant difference in the number of anti-glaucoma medications was found between Phaco-ExPRESS and Phaco-Trab (*P* = 0.86). However, Phaco-ExPRESS showed a significant decrease in the number of anti-glaucoma medications and patients taking anti-glaucoma medications when compared with Phaco-Trab (*P* < 0.01). Moreover, higher complete success rate was shown in Phaco-ExPRESS at 12 months postoperatively (*P* < 0.01), but not in qualified success rate (*P* = 0.24). The Kaplan-Meier survival analysis of both groups are illustrated in Fig. 2, highlighting significant differences of cumulative probability of success in both qualified and complete success.

**Table 2:** Preoperative and postoperative change of intraocular pressure (IOP) and anti-glaucoma medication

	Phaco-ExPRESS (n = 33)	Phaco-Trab (n = 34)	*P* value
IOP (mmHg)			
PRE-OP	21.61 ± 5.72	21.60 ± 3.49	0.15
POST-OP 2 W	9.21 ± 2.59	10.91 ± 2.49	**0.01**
POST-OP 1 M	10.57 ± 2.58	12.08 ± 3.03	**0.04**
POST-OP 3 M	12.29 ± 3.03	12.54 ± 3.87	0.78
POST-OP 6 M	12.05 ± 3.01	12.52 ± 3.61	0.58
POST-OP 12 M	12.57 ± 2.81	13.29 ± 3.67	0.38
No. of anti-glaucoma medications at baseline	2.42 ± 1.20	2.47 ± 0.86	0.86
No. of anti-glaucoma medications at 12 M	0.24 ± 0.56	1.02 ± 1.08	** < 0.01**
No. of patients taking anti-glaucoma medications at 12 M	5	20	** < 0.01**
No. of patients achieved qualified success at 12 M	33 (100.0%)	31 (91.2%)	0.24
No. of patients achieved complete success at 12 M	28 (84.9%)	14 (41.2%)	** < 0.01**

*PRE-OP=* preoperative; *POST-OP= *postoperative; *W*= week; *M*= month. Bold font indicates signifcance of *P *< 0.05

### Microstructural changes of the filtering blebs after treatments

**Fig. 3:**
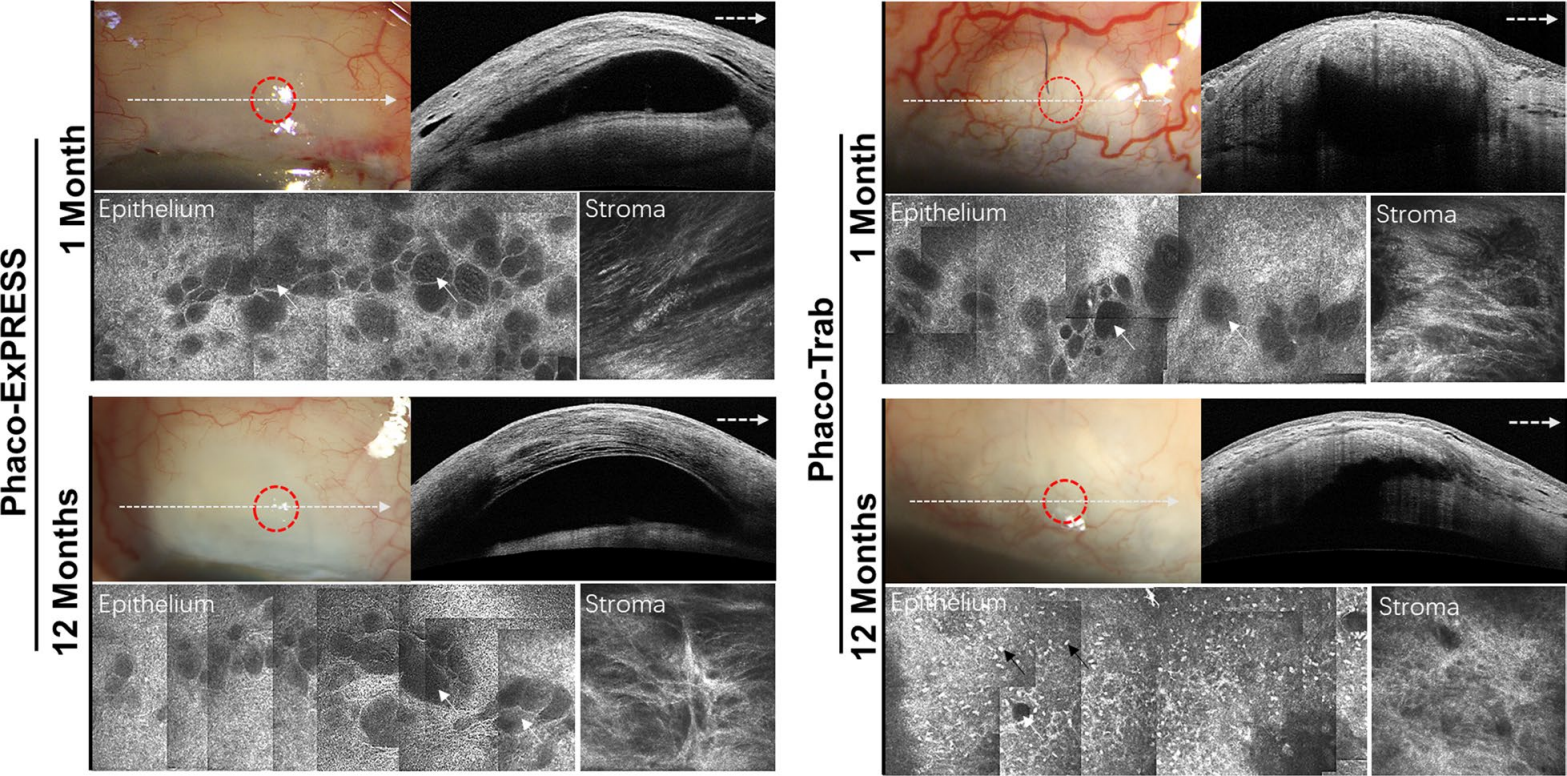
Filtering bleb follow-up of Phaco-ExPRESS and Phaco-Trab group. Morphology of filtering bleb of Phaco-ExPRESS at 1 month and 12 months (left). Morphology of filtering bleb after Phaco-Trab at the same time points (right). As shown in the slit-lamp photography, an anterior segment optical coherence tomography (AS-OCT) was taken through the white arrow line across the filtering bleb and in vivo confocal microscopy (IVCM) scanning was taken at the highest point of filtering bleb, in which epithelium and stroma of conjunctiva, circled by red dotted line, were scanned. In both groups, microcysts (showed by white arrow) and loose connective tissue can be seen at 1 month postoperatively. However, in Phaco-ExPRESS, the height and morphology of filtering bleb under AS-OCT did not change significantly at 12 months, while Phaco-Trab demonstrated a decrease in filtering bleb height and thickening of filtering bleb wall. In Phaco-ExPRESS, the epithelial microcysts area at the same region did not change at 12 months, while epithelial microcysts in Phaco-Trab decreased significantly. Phaco-Trab showed increased epithelial hyperreflective dot (black arrows) density at 12 months while no hyperreflective dot were discovered in Phaco-ExPRESS at the same time point

**Fig. 4:**
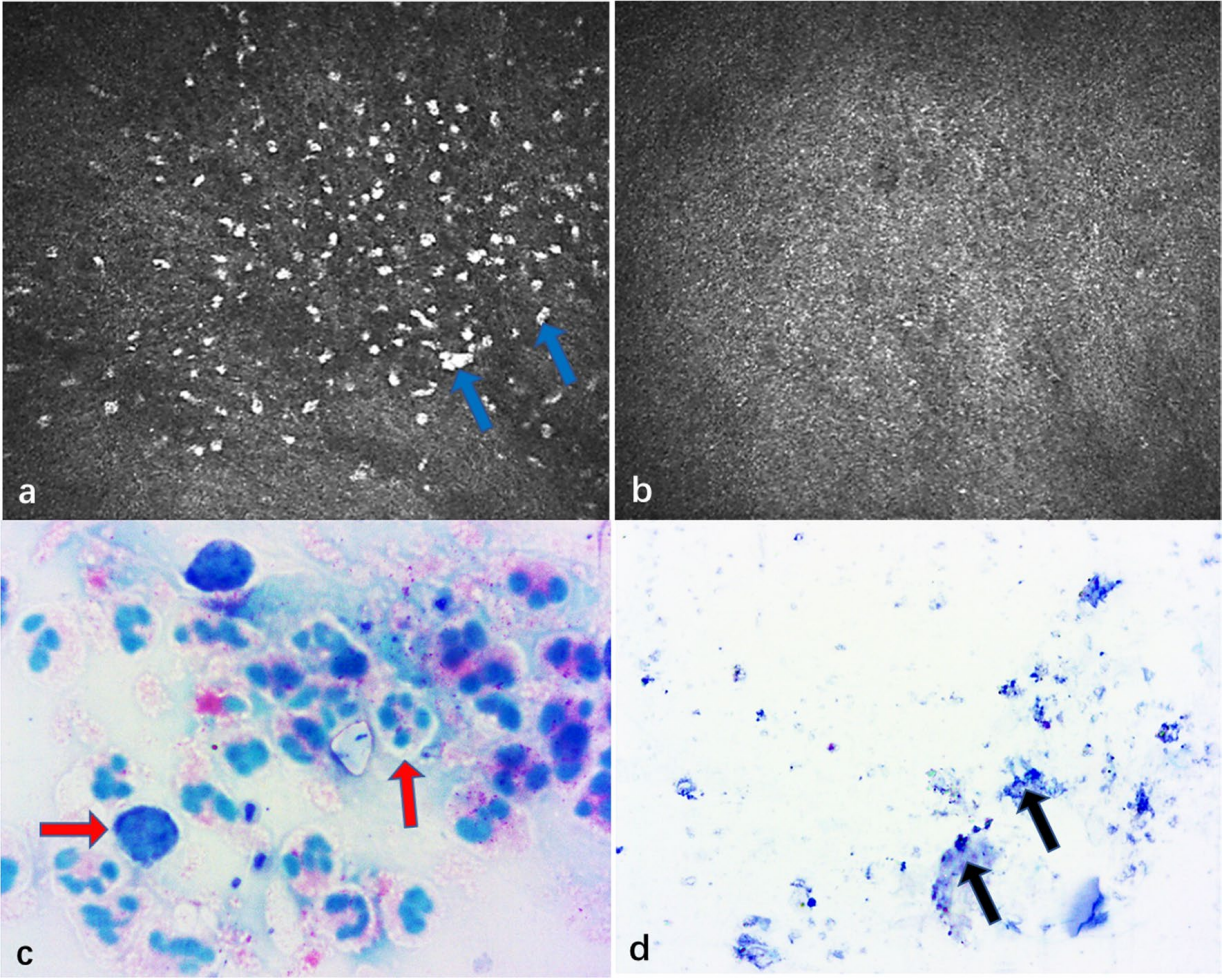
Giemsa stain of hyperreflective dot. In vivo confocal microscopy (IVCM) and corresponding Giemsa stain of two different patients are shown. In **a** and **c**, the patient whose IVCM showed numerous hyperreflective dots (blue arrows) in the conjunctiva also showed numerous neutrophil- and monocyte-like inflammatory cells (red arrows) in a scrape sample of conjunctiva over filtering bleb. In **b** and **d**, the patient showed no hyperreflective dots in the IVCM and likewise saw no appearance of inflammatory cells but only fragments of cells (black arrows) from the scrape sample

At one month after treatment with Phaco-ExPRESS (Fig. 3a), there were numerous microcysts with oval-shaped extracellular structures in the epithelium of the filtering bleb. These microcysts were ranging from 10 to 90 μm and located at the intermediate layer of the epithelium (10–30 μm). In the stroma of the bleb (60–80 μm), the stripping phenomenon was obvious in AS-OCT in response to the stroma. At 12-month follow-up for Phaco-ExPRESS, these microcysts became bigger in 87.9% of filtering blebs and stripping phenomenon remained in 69.7% of filtering blebs. However, in Phaco-Trab (Fig. 3b), the number of epithelial microcysts was decreased in 82.4% of filtering blebs and the stripping phenomenon was only present in 38.2% of filtering blebs with hyperreflective subepithelial mass in AS-OCT. Notably, at 12 months postoperatively, the epithelial microcysts disappeared and were replaced with many hyperreflective dots in 61.8% of filtering blebs in the Phaco-Trab group. However, the hyperreflective dots were rarely observed in the group of Phaco-ExPRESS, in which only 3.0% showed increased hyperreflective dots at 12 months postoperatively. To specify the characteristics of hyperreflective dots, we took some conjunctival scraping specimens over the filtering bleb and confirmed that the hyperreflective dots were neutrophil- and monocyte-like cells (Fig. 4).

**Fig. 5:**
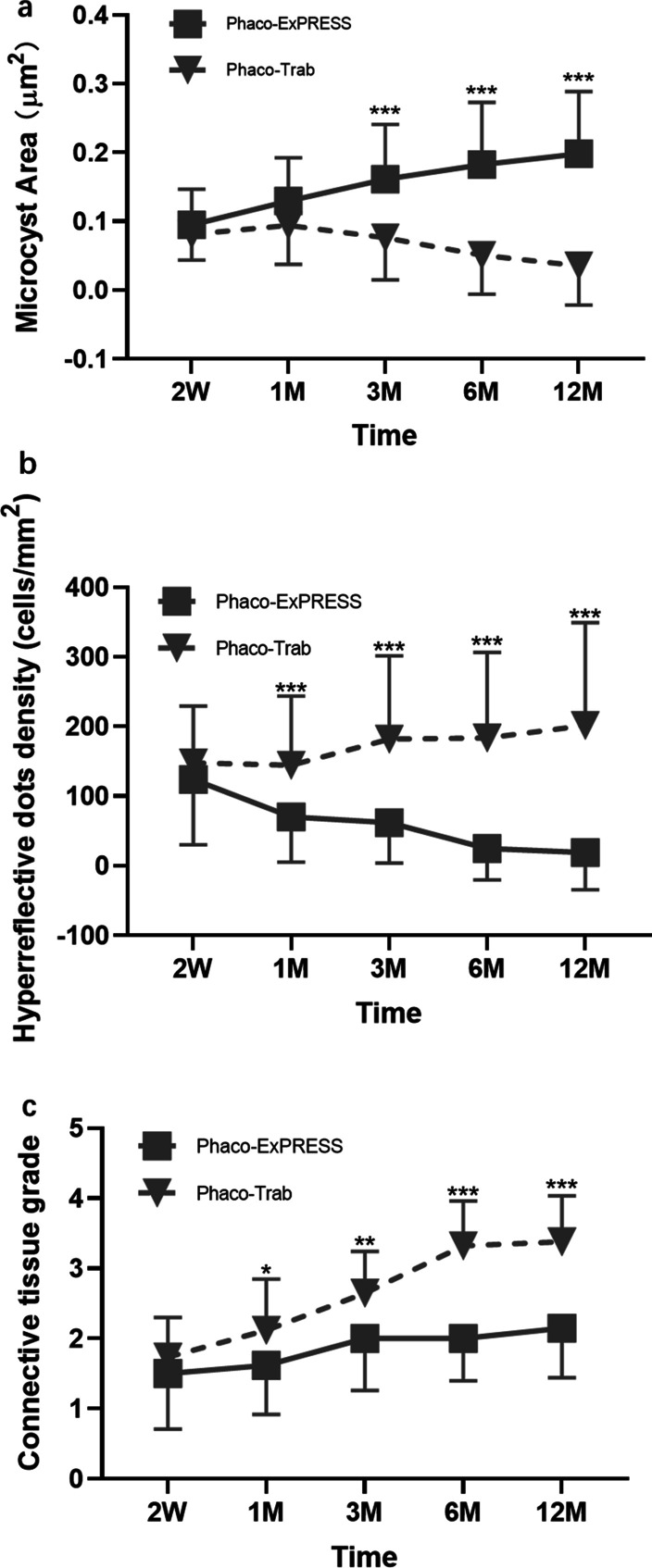
In vivo confocal microscopy (IVCM) parameters follow-up. IVCM parameters follow-up of postoperative 2 weeks (2 W), 1 month (1 M), 3 months (3 M), 6 months (6 M), and 12 months (12 M) are shown. *P* < 0.05 is defined as statistically significant between the two groups at the same time point (*), *P* < 0.01 is defined as highly significant (**) and *P* < 0.001 is defined as extremely significant (***). **a** Significantly higher mean microcysts area in Phaco-ExPRESS at 3 M, 6 M, and 12 M postoperatively (*P* < 0.001). **b** Significantly lower hyperreflective dot density in Phaco-Trab at 1 M, 3 M, 6 M, and 12 M postoperatively when compared with the other group (*P* < 0.001). **c** Significantly higher connective tissue grade in Phaco-Trab at 1 M, 3 M, 6 M, and 12 M postoperatively (*P* = 0.01 at 1 M, *P* = 0.001 at 3 M and *P* < 0.001 at 6 M and 12 M)

Overall, in the one-year follow up, the mean area of epithelial microcysts was significantly increased from 0.10 ± 0.05 to 0.20 ± 0.09 μm^2^ per μm^2^ in the Phaco-ExPRESS group, but decreased from 0.08 ± 0.04 to 0.04 ± 0.06 μm^2^ per μm^2^ in the Phaco-Trab group (Fig. 5a) from postoperative 2 weeks to 12 months. On the contrary, the hyperreflective dots increased by 36.3% in Phaco-Trab and decreased by 84.9% in Phaco-ExPRESS (Fig. 5b). In the stroma of the filtering blebs, a loose connective tissue network (connective tissue grade ≤ 2) was maintained in 26 eyes (78.8%) at 12 months in Phaco-ExPRESS but only observed in 3 eyes (8.8%) in Phaco-Trab (Fig. 5c). Moreover, the density of hyperreflective dots was negatively correlated with the microcysts area (r = − 0.7, *P* < 0.01) while positively associated with the connective tissue grade (r = 0.5, *P* < 0.01). There was also a negative association between the microcysts area and connective tissue grade (r = − 0.7, *P* < 0.01). As shown in Additional file 2: Table S1, the Spearman ρ test revealed that the correlation between the 12 months postoperative anti-glaucoma medications and 12 months hyperreflective cell density was significant (r = 0.4, *P* < 0.01), whereas it did not reach statistical significance for preoperative anti-glaucoma medications and 12 months hyperreflective cell density (r = 0.2, *P* = 0.10).

To determine interobserver variability, both groups of patients were combined. The ICC of epithelial microcysts, hyperreflective dots and connective tissue grade estimated by investigator 1 and investigator 2 were 0.753, 0.791 and 0.834, respectively.

### Complications and additional procedures

The types of complications were similar in both groups (*P* > 0.05). No serous choroidal detachment and corneal decompensation was found in both groups. The postoperative endothelial cell count at 12 months was 1752.08 ± 240.21 cells/mm^2^ in Phaco-ExPRESS and 1735.01 ± 200.12 cells/mm^2^ in Phaco-Trab (*P* = 0.43). Conjunctival wound dehiscence was found in five patients (15.2%) in Phaco-ExPRESS and three patients (8.8%) in Phaco-Trab in the early postoperative period. These patients required wound repair by suturing. Shallow anterior chamber was found in four patients in Phaco-ExPRESS and two patients in Phaco-Trab. All of them recovered after conservative treatment. Additionally, laser suture lysis was used in two patients from Phaco-ExPRESS and seven patients from Phaco-Trab. Postoperative needling with intra-bleb 5-FU was performed in two patients in Phaco-ExPRESS and four patients in Phaco-Trab. No additional glaucoma surgery was conducted in both groups.

## Discussion

The development of cataract surgery technique has made combined surgery an attractive option for the patients with coincident POAG and cataract [1]. However, trabeculectomy alone can produce 50% of the complete success rate at 5 years postoperatively, but it was reduced to 19% in combined surgery Phaco-Trab [25]. It was possible that the combination surgery intensified postoperative inflammation to accelerate the fibrosis of filtering bleb leading to unsatisfied IOP control [8, 9]. In contrast, Ex-PRESS glaucoma minishunt implantation was found to be effective in reducing the postoperative inflammation of filtering surgery and obtaining high success rate [26–30]. In current study, we also found Phaco-ExPRESS reduced the number of anti-glaucoma medications (0.24 ± 0.56 vs. 1.02 ± 1.08) and had higher complete success rate than did by Phaco-Trab (84.9% vs*.* 41.2%), although no statistically significant difference in the qualified success rate was observed between the two groups at 12 months postoperatively. Therefore, for patients with coexisting POAG and cataract, phacoemulsification combined with Ex-PRESS rather than trabeculectomy could provide better IOP control and less postoperative anti-glaucoma medications. Notably, no serous choroidal detachment and corneal decompensation was found in Phaco-ExPRESS and Phaco-Trab. The number of corneal endothelial cells were similar between the two groups at the 12-month visit, indicating that Phaco-ExPRESS or Phaco-Trab were safe for the patients.

As the epithelial microcystic spaces are thought to be potential channels for passage of aqueous humor after filtering surgery and presumed to be a positive predictive factor for functioning blebs [17, 22, 23, 31], we further evaluated the microstructural changes of filtering blebs by slit-lamp, in vivo confocal microscope and AS-OCT. In the functioning bleb (Fig. 1a), we found that there were numerous microcysts accompanied with loose fibrous network. However, in the nonfunctioning bleb (Fig. 1b), the epithelial microcysts were absent or few and the subepithelial connective tissue became dense. These results were in consistent with a previous study showing that functioning blebs had numerous intraepithelial optically-empty microcysts and loosely arranged subepithelial connective tissues [17]. Quantitative analysis of the microcysts area further demonstrated that at 12-month after Phaco-ExPRESS, increased microcysts area was observed in 87.9% of filtering blebs, whereas 82.4% of the blebs showed a decrease in microcysts in Phaco-Trab. We also found obvious striping phenomenon within the bleb wall in Phaco-ExPRESS, but it was rarely detected in the blebs of Phaco-Trab. These results strongly support that the filtering blebs created by Phaco-ExPRESS rather than Phaco-Trab were more likely to form a functioning bleb with better control of the IOP.

It has been shown that the most important cause leading to failure of the filtering bleb is unduly marked or persistent inflammation with deposition of fibrous tissue in the subconjunctiva [32]. Currently, we found that the hyperreflective dots that identified as neutrophil- and monocyte-like cells were decreased gradually from 1 to 12 months in 97.0% of filtering blebs in Phaco-ExPRESS. In contrast, these cells were increased in 61.8% of filtering blebs in Phaco-Trab. These inflammatory cells in the subconjunctiva may lead to apoptosis of the goblet cells around the microcysts, resulting in a decreased area of microcysts but increased fibrosis of filtering bleb [33]. Our results further showed that the density of the hyperreflective dots was negatively associated with the area of epithelial microcysts but positively correlated with the grade of subepithelial connective tissue. Moreover, it was proposed that the Ex-PRESS implantation might induce less inflammation in filtering blebs as devoid of inflammatory cells and less bleb vascularity were observed [13, 34]. It thus provided additional evidence that a functioning bleb was more likely to be created by Phaco-ExPRESS. Even though the Spearman ρ test revealed that the inflammatory cell density at 12 months was correlated with the number of postoperative anti-glaucoma medications, a prospective study with larger sample size is needed to further delineate the relationship between anti-glaucoma medications and postoperative inflammation. Overall, implantation of the Ex-PRESS might be more preferred than trabeculectomy when combined with phacoemulsification for the treatment of patients with coincident POAG and cataract.

Regarding cost-effectiveness of glaucoma surgery, a previous study showed that conventional glaucoma surgery was more cost-efficient in lowering IOP compared with various minimally invasive glaucoma surgeries [35], but in patients with coexisting glaucoma and cataract, the cost-effectiveness for microshunt implantation versus standard trabeculectomy when combined with cataract surgery is inconclusive. Sood et al. showed that the implantation of Hydrus Microstent or iStent inject during cataract surgery is cost-effective compared with cataract surgery alone within 5 years, demonstrating that the combination of minimally invasive glaucoma surgeries and cataract surgery may be cost-effective in managing patients with coexisting glaucoma and cataract [36]. Indeed, more randomized controlled trials for cost-effectiveness analysis of Phaco-ExPRESS and Phaco-Trab are needed to compare their cost-effectiveness specifically.

This study is limited by its retrospective design. A prospective investigation with larger sample size is needed to verify our findings in Chinese and other populations. Moreover, although the patients have been treated by Phaco-ExPRESS or Phaco-Trab, some may require additional anti-glaucoma medications to reach the target IOP. These medications such as prostaglandin could increase the inflammation of the filtering blebs that may affect the outcome of the success rate.

## Conclusions

In conclusion, both Phaco-ExPRESS and Phaco-Trab are equally effective in managing POAG. However, Phaco-ExPRESS generates a better filtering bleb with larger microcysts area, looser connective tissue, and less inflammation than that of Phaco-Trab, suggesting that Phaco-ExPRESS could be more potent than Phaco-Trab for the treatment of patients with coincident POAG and cataract.

## Supplementary Information

**Additional file 1: Fig. S1.** Connective tissue grading sample. a. Picture of grade 1 connective tissue with an average gray value less than 90.00. b. Mild reflectivity (grade 2) connective tissue with gray value between 90.01 to 105.00. c. Connective tissue with moderate reflectivity (grade 3) defined as gray value between 105.01 to 125.00. d. High reflectivity (grade 4) connective tissue with gray value greater than 125.01.

**Additional file 2: Table S1.** Correlation between 12-month (12 M) hyperreflective dot density and number of anti-glaucoma medications.

## Data Availability

The datasets used and/or analyzed during the current study are available from the corresponding author upon reasonable request.
