# Supplementary material for: Comparison of Ex-PRESS implantation versus trabeculectomy combined with phacoemulsification in primary open-angle glaucoma: a retrospective in vivo confocal microscopy study
Source: Eye Vis (Lond). 2022 Feb 10;9:7. doi: 10.1186/s40662-022-00278-2 (PMC8841063; doi:10.1186/s40662-022-00278-2)
Supplement: Supplementary file 1 — Additional file 1: Fig. S1. Connective tissue grading sample. a. Picture of grade 1 connective tissue with an average gray value less than 90.00. b. Mild reflectivity (grade 2) connective tissue with gray value between 90.01 to 105.00. c. Connective tissue with moderate reflectivity (grade 3) defined as gray value between 105.01 to 125.00. d. High reflectivity (grade 4) connective tissue with gray value greater than 125.01. [file 40662_2022_278_MOESM1_ESM.docx]

**Supplementary figure 1: Connective tissue grading sample**
