# Supplementary material for: Comparison of Ex-PRESS implantation versus trabeculectomy combined with phacoemulsification in primary open-angle glaucoma: a retrospective in vivo confocal microscopy study
Source: Eye Vis (Lond). 2022 Feb 10;9:7. doi: 10.1186/s40662-022-00278-2 (PMC8841063; doi:10.1186/s40662-022-00278-2)
Supplement: Supplementary file 2 — Additional file 2: Table S1. Correlation between 12-month (12 M) hyperreflective dot density and number of anti-glaucoma medications. [file 40662_2022_278_MOESM2_ESM.docx]

**Supplementary Table 1: Correlation between 12-month (12M) hyperreflective dot density and number of anti-glaucoma medications.**

|  | **Preoperative anti-glaucoma medications**  **(r, *P* value)** | **12M anti-glaucoma medications**  **(r, *P* value)** |
| --- | --- | --- |
| **12M hyperreflective dot count (cells/mm^2^)** | 0.20, 0.10 | 0.44, <0.01 |

Spearman ρ test showed that the number of 12M anti-glaucoma medications is positively correlated with 12M hyperreflective dot density (r = 0.44, *P* < 0.01).
